# Cytotoxic and Genotoxic Effects of Fluconazole on African Green Monkey Kidney (Vero) Cell Line

**DOI:** 10.1155/2018/6271547

**Published:** 2018-11-01

**Authors:** Regianne Maciel dos Santos Correa, Tatiane Cristina Mota, Adriana Costa Guimarães, Laís Teixeira Bonfim, Rommel Rodriguez Burbano, Marcelo de Oliveira Bahia

**Affiliations:** Laboratório de Citogenética Humana, Instituto de Ciências Biológicas, Universidade Federal do Pará, Belém, Pará, Brazil

## Abstract

Fluconazole is a broad-spectrum triazole antifungal that is well-established as the first-line treatment for* Candida albicans* infections. Despite its extensive use, reports on its genotoxic/mutagenic effects are controversial; therefore, further studies are needed to better clarify such effects. African green monkey kidney (Vero) cells were exposed* in vitro* to different concentrations of fluconazole and were then evaluated for different parameters, such as cytotoxicity (MTT/cell death by fluorescent dyes), genotoxicity/mutagenicity (comet assay/micronucleus test), and induction of oxidative stress (DCFH-DA assay). Fluconazole was used at concentrations of 81.6, 163.2, 326.5, 653, 1306, and 2612.1*μ*M for the MTT assay and 81.6, 326.5, and 1306*μ*M for the remaining assays. MTT results showed that cell viability reduced upon exposure to fluconazole concentration of 1306*μ*M (85.93%), being statistically significant (P<0.05) at fluconazole concentration of 2612.1*μ*M (35.25%), as compared with the control (100%). Fluconazole also induced necrosis (P<0.05) in Vero cell line when cells were exposed to all concentrations (81.6, 326.5, and 1306*μ*M) for both tested harvest times (24 and 48 h) as compared with the negative control. Regarding genotoxicity/mutagenicity, results showed fluconazole to increase significantly (P<0.05) DNA damage index, as assessed by comet assay, at 1306*μ*M versus the negative control (DI=1.17 vs DI=0.28, respectively). Micronucleus frequency also increased until reaching statistical significance (P<0.05) at 1306*μ*M fluconazole (with 42MN/1000 binucleated cells) as compared to the negative control (13MN/1000 binucleated cells). Finally, significant formation of reactive oxygen species (P<0.05) was observed at 1306*μ*M fluconazole vs the negative control (OD=40.9 vs OD=32.3, respectively). Our experiments showed that fluconazole is cytotoxic and genotoxic in the assessed conditions. It is likely that such effects may be due to the oxidative properties of fluconazole and/or the presence of FMO (flavin-containing monooxygenase) in Vero cells.

## 1. Introduction

Fluconazole is a broad-spectrum triazole antifungal drug and is, therefore, used to treat infections caused by various pathogenic fungi [1]. Its mechanism of action is based on inhibition of the oxidative enzyme lanosterol 14-*α*-demethylase, which is associated with cytochrome P450 and is essential in the bioregulation of fluidity, asymmetry, and integrity of the cellular membrane [2]. It is well-established as the first line of treatment for systemic* Candida albicans* infections [3], and it is, hence, an important drug in the areas of obstetrics and gynecology for the treatment of vaginal candidiasis. It is also used in patients with compromised immunity, such as those with acquired immunodeficiency syndrome and those with neutropenia due to chemotherapy for cancer. Such patients are at risk of developing* Candida albicans* infection, which can progress into a systemic infection [4, 5]. Despite its importance, its teratogenic effects in newborns, embryotoxicity in animals after drug administration, and passage into breast milk have been reported [2, 6, 7].

Reports on the genotoxic/mutagenic effects of fluconazole are controversial. For example, fluconazole did not increase the frequency of chromosomal aberrations in rat's bone marrow* in vivo*. However, in* in vitro* test, it induced significantly high frequencies of chromosomal aberrations, sister-chromatid exchanges, and micronuclei formation in peripheral blood lymphocytes [8].

Given the scarcity of studies dealing with the genotoxicity of fluconazole, together with the need to study these effects in different test systems [8], we decided to increase the existing knowledge by evaluating the genotoxic effects of fluconazole allied to parameters, such as cytotoxicity and induction of oxidative stress on an African green monkey kidney (Vero) cell line, through comet and micronuclei assays.

## 2. Materials and Methods

### 2.1. Chemical Compounds

**Figure 1:**
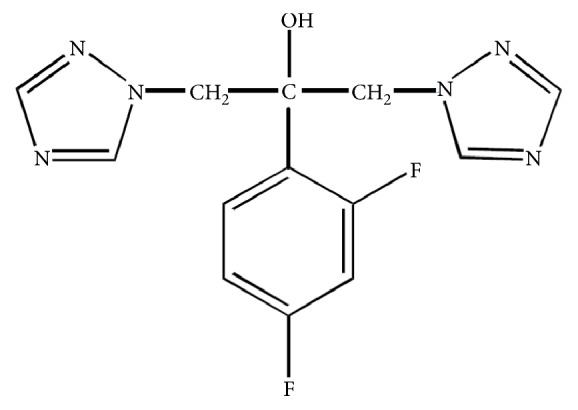
Chemical structure of fluconazole.

Pure-grade fluconazole (CAS: 86386-73-4) was purchased from Sigma Chemical Co. (St. Louis, MO, USA). The chemical structure of fluconazole (2-(2,4-difluorophenyl)-1,3-bis(1H-1,2,4-triazol-1-yl)-2-propanol) is presented in Figure 1. The drug was dissolved in pure-grade dimethylsulfoxide (DMSO) (CAS 67-68-5, Sigma Chemical Co, St. Louis, MO, USA) to achieve a less than 1% (v/v) DMSO final concentration in the cultures. N-methyl-N-nitrosourea (NMU) (CAS 684-93-5) was purchased from Sigma Chemical Co. (St. Louis, MO, USA) and was diluted in DMSO as well.

### 2.2. Cell Culture

The Vero cell line was commercially obtained from Rio de Janeiro, Brazil cell bank. Vero cells are isolated from kidney epithelial cells of the African green monkey [9]. These cells were grown in Dulbecco's modified eagle's medium (Sigma Chemical Co., St. Louis, MO, USA) supplemented with 15% fetal bovine serum (Gibco, Grand Island, NY, USA), streptomycin (0.1mg ml-1), and penicillin (99 Uml-1) and were kept in an incubator at 37°C and 5% CO2. Cells were subcultured two or three times a week.

### 2.3. MTT Assay

For the MTT (3-(4,5-dimethylthiazol-2-yl)-2,5-diphenyltetrazolium bromide) assay, Vero cells were grown in 96-well culture plates at a concentration of 0.008 × 10^6^ cells/well and were incubated for 24 hours. After the initial period of incubation, cells were treated with different concentrations of fluconazole for 24 hours. Then, 100 *μ*l of MTT (5000*μ*g/mL) was added to the cells for 3 hours. Next, the MTT was removed, and 100 *μ*l of DMSO (Sigma®) was added for 1 hour to dissolve the formazan formed during the process. Afterwards, DMSO was measured by spectrophotometry (*λ*=562nm). Cell survival was calculated as the absorbance percentage compared to the control absorbance. The fluconazole concentrations used in the experiment were 81.6, 163.2, 326.5, 653, 1306, and 2612.1*μ*M. These concentrations were chosen based on previous experiments carried out* in vitro* [8]. However, clinical studies showed that the maximum fluconazole concentration observed in volunteers was tenfold lower than the lowest concentration (81.6*μ*M) used in the present study [10].

### 2.4. Micronucleus Test

Vero cells were treated with fluconazole for 24 h in 25-cm^2^ sterile flasks (Corning) at a concentration of 1x10^6^ cells/mL. After treatment, 3-*μ*g/mL cytochalasin B (Sigma Chemical Co.) was added for another 24 hours at 37°C. The cells were harvested, centrifuged for 5 minutes at 800 rpm, and treated with 5-mL hypotonic solution (KCl 0.075 M). Afterwards, the cells were washed once with 5-mL 5:1 (v/v) and twice with 5-mL 3:1 (v/v) cold methanol:acetic acid solution. The slides were prepared and stained with 5% Giemsa dye (Sigma Chemical Co.) in phosphate buffer solution (PBS), pH 6.8, for 5 minutes. Micronuclei (MN) were scored in 1000 binucleated cells using the criteria adopted from the study by Fenech et al. [11]. The frequency of binucleated cells containing one or more MN was also determined. As a measure of cytotoxicity, the cytokinesis-block proliferating index (CBPI) was calculated according to the following formula: CBPI = [M1 + 2(M2) + 3(M3) + 4(M4)]/N, where M1-M4 represents the number of cells with 1-4 nuclei per 500 cells. As previously mentioned, the fluconazole concentrations used were 81.6, 326.5, and 1306*μ*M. NMU, which is a known carcinogenic alkylating agent, was used as the positive control, with final concentration of 1212.6*μ*M. The single NMU concentration was defined according to previous MTT assays performed in our laboratory (data not shown).

### 2.5. Comet Assay (Alkaline Version)

For the alkaline version of the comet assay, Vero cells were grown in 25-cm^2^ sterile flasks (Corning) at a concentration of 1x10^6^ cells/mL and were treated with different concentrations of fluconazole (81.6, 326.5, and 1306*μ*M) for 3 hours. NMU (1212.6*μ*M) was used as the positive control. After treatment, 450 *μ*L of the cell suspension was homogenized with 300 *μ*L of a low-melting-point agarose (0.8%). The cell suspension was spread onto microscope slides precoated with a normal-melting-point agarose (1.5%) and was covered with a coverslip (24x60mm). After 5 minutes at 4°C, the coverslip was removed, and the slides were immersed in cold lysis solution (2.5M NaCl; 100 mM EDTA; 10 mM Tris, 10% DMSO, and 1% Triton-X; pH=10). After lysis, the slides were placed in an electrophoresis chamber and were covered with freshly made electrophoresis buffer (300mM NaOH and 1 mM EDTA; pH>13). The electrophoresis was run for 25 minutes (34V and 300 mA). Afterwards, the slides were neutralized through submersion in distilled water (4°C) for 5 minutes and were fixed in 100% ethanol for 3 minutes. The slides were stained with 20-*μ*g/mL ethidium bromide immediately prior to analyses and were prepared in duplicate. One-hundred cells, or 50 cells from each slide, were screened per sample using a fluorescence microscope (Olympus BX41) at 40x magnification. The DNA damage index (DI), or the relative intensity of fluorescence in the comet's tail with regard to frequency of DNA breaks, was visually determined, and the following five categories (0-4) were used: class 0 (no damage); class 1 (little damage with tail length shorter than the nucleus diameter); class 2 (medium damage with tail length one or two times greater than the nucleus diameter); class 3 (significant damage with tail length one or two times greater than the nucleus diameter); and class 4 (significant damage with tail length three times greater than the nucleus diameter). Moreover, DI was determined by the following formula:

(1) $$\begin{array}{c} DI\;\left(\;au\;\right)\text{: }\frac{\left[\left(N1*1+N2*2+N3*3+N4*4\right)\right]}{100\;\left(\text{total number of analyzed cells}\right)} \end{array}$$

where DI is DNA damage index, au is arbitrary unit, and N1-N4 are cells in classes 1, 2, 3, and 4.

### 2.6. Dichlorodihydrofluorescein Diacetate (DCFH-DA) Assay

Intracellular reactive oxygen species (ROS) generation was evaluated using the fluorescent probe dichlorodihydrofluorescein diacetate (DCFH-DA) (Sigma Chemical Co., St. Louis, MO, USA). Vero cells were grown in sterile 6-well culture plates (Corning) (0.5x10^6^ cells/well) and were exposed to fluconazole at different concentrations (81.6, 326.5 and 1306*μ*M) for 1 hour at 37°C. Thereafter, the cells were collected by centrifugation and were washed with PBS at 1000 rpm for 5 minutes. After the second centrifugation, the cells were suspended in PBS, and DCFH-DA was added to a final concentration of 10*μ*M. The suspension was incubated in the dark for 30 minutes at 37°C. After another washing with PBS, the samples were analyzed by spectrophotometry with an emission wavelength of 528 nm and an excitation wavelength of 485 nm. Hydrogen peroxide (2mM) was used as the positive control.

### 2.7. Evaluation of Necrosis and Apoptosis by Fluorescent Differential Staining with Hoechst 33342/Propidium Iodide (PI)/Fluorescein Diacetate (FDA)

To evaluate apoptosis and necrosis of Vero cells, 0.5 × 10^6^ cells were seeded in 6-well culture plates (Corning) with complete medium. After 24 hours, the cells were treated with different concentrations of fluconazole (81.6, 326.5, and 1306*μ*M) for 24 and 48 hours. NMU (1.212.6*μ*M) was used as the positive control. The cells were then trypsinized, and 100 *μ*L of cell suspension was mixed with a previously prepared solution of Hoechst 33342/Propidium Iodide (PI)/Fluorescein Diacetate (FDA) (100*μ*L dying solution of 25 *μ*L PI 1 mg/ml in distilled water + 50 *μ*L FAD in 1.5 mg/mL DMSO + 10 *μ*L Hoechst 33342 [HO] in 1 mg/mL distilled water + 15 *μ*L PBS [pH= 8.0]) after centrifugation. The cells were incubated with dyes for 5 minutes in bath water and were subsequently analyzed using an Olympus BX41 fluorescence microscope with triple filter DAPI/FITC/TRITC [DAPI (4′,6-diamidino-2-phenylindole); FITC (fluorescein isothiocyanate); TRITC (tetramethylrhodamine-5-(and 6)-isothiocyanate)]. Three hundred cells were analyzed for each treatment group according to the criteria used by Hashimoto et al. [12].

### 2.8. Statistical Analysis

Statistical analysis was performed by using the BIOESTAT 5.0 software with* P* values<0.05 considered significant [13]. For parametric data sets, statistical analysis was performed using ANOVA, followed by the Tukey test. For nonparametric data sets, we used Kruskal–Wallis test followed by Dunn test.

## 3. Results

### 3.1. MTT Assay

**Figure 2:**
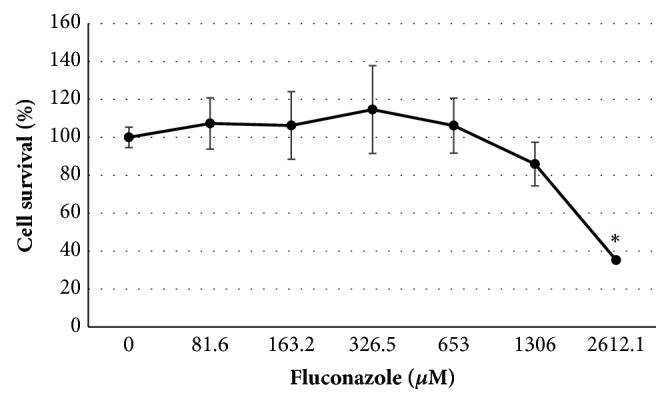
Effects of fluconazole in Vero cells analyzed by MTT assay. *∗*P<0.05 (Kruskal–Wallis/Dunn posttest) when compared with control. Data are expressed as the mean values obtained from three experiments in duplicate.

The results of MTT assay, which was assessed 24 hours after treatment with fluconazole, demonstrated a decrease in the survival percentages upon exposure to fluconazole concentration of 1306*μ*M (85.93%); such decrease in survival was found to be statistically significant at fluconazole concentration of 2612.1*μ*M (35.25%), as compared with the control (100%). In addition, significant differences were observed in the following comparisons: 81.6*μ*M (107.34%) vs 2612.1*μ*M (35.25%); 163.2*μ*M (106.27%) vs 2612.1*μ*M (35.25%); 326.5*μ*M (114.68%) vs 2612.1*μ*M (35.25%); and 652.8*μ*M (106.19%) vs 2612.1*μ*M (35.25%) (Figure 2).

### 3.2. Micronucleus Test

**Figure 3:**
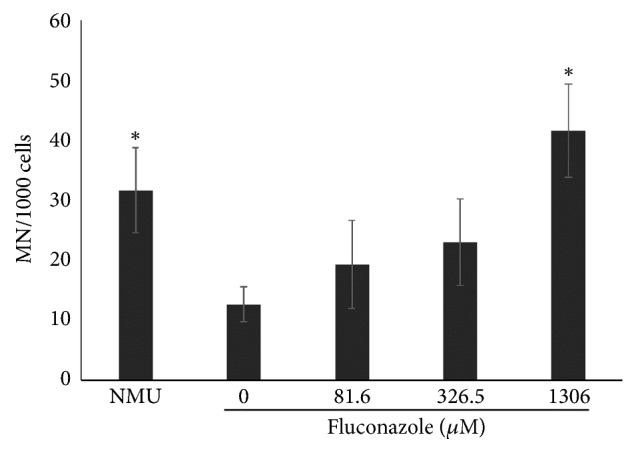
Micronucleus frequency observed in Vero cell line after exposition to different concentrations of fluconazole for 24 h. *∗*P < 0.05 related to control (ANOVA/Tukey posttest). Data are expressed as the mean values obtained from three experiments.

MN tests showed an increase in the frequency of micronuclei induced by fluconazole. For every 1000 binucleated cells analyzed at fluconazole concentrations of 81.6, 326.5, and 1306*μ*M, the corresponding mean frequencies of MN were 19, 23, and 42, respectively. Statistical significance (P<0.05) was observed at 1306*μ*M fluconazole as compared to the negative control, with 42 MN/1000 and 13 MN/1000 binucleated cells, respectively. NMU-treated cells demonstrated a mean frequency of 32 MN/1000 binucleated cells, which was also statistically significant (P<0.05) as compared to the negative control (Figure 3). CBPI mean frequencies did not significantly differ between the negative control and the treated groups (Table 1). The concentrations used in the MN, comet, and DCFH-DA assays and in the evaluation of necrosis and apoptosis by fluorescent differential staining were defined based on the results of cell viability. The chosen concentrations had a viability percentage greater than 50% as compared with that of the control.

### 3.3. Comet Assay (Alkaline Version)

**Figure 4:**
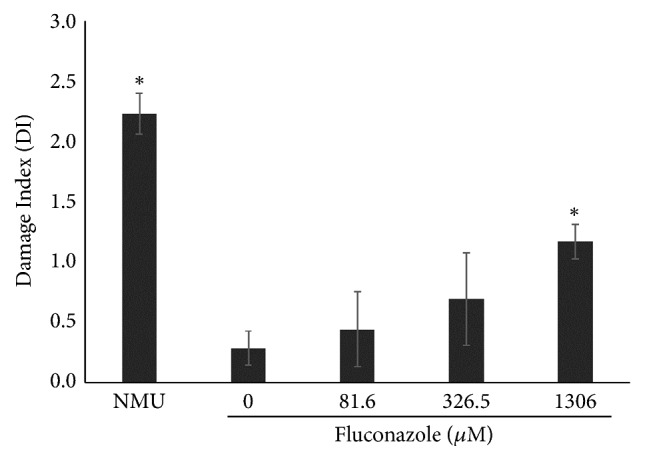
Effects of fluconazole in Vero cell line analyzed by comet assay. *∗*P<0.05 (ANOVA/Tukey posttest) when compared with control. Data are expressed as the mean values obtained from three experiments.

The results of comet assay, which was assessed after treatment with fluconazole, showed a dose-dependent increase in DNA DI of Vero cell line. For every 100 cells analyzed at fluconazole concentrations of 81.6, 326.5, and 1306*μ*M, the corresponding DIs were 0.44, 0.69, and 1.17, respectively. Statistical significance (P<0.05) was observed at 1306*μ*M versus the negative control (DI=1.17 vs DI=0.28, respectively). NMU-treated cells showed a DI=2.23, which was also statistically significant (P<0.05) when compared to that of the negative control. Furthermore, the DI of NMU-treated cells was also statistically significant (P < 0.05) when compared to all concentrations of fluconazole (Figure 4).

### 3.4. ROS Generation

**Figure 5:**
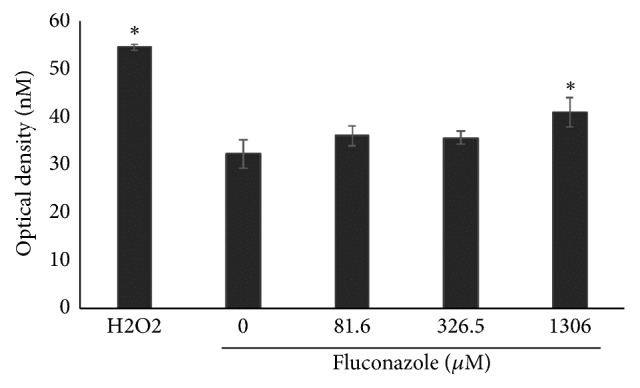
ROS generation induced by fluconazole in Vero cell line. *∗*P<0.05 (ANOVA/Tukey posttest) when compared with control. Data are expressed as the mean values obtained from three experiments.

ROS generation was assessed after treatment with fluconazole by DCFH-DA assay, and optical density (OD) means of 36.1, 35.7, and 40.9 were observed at fluconazole concentrations of 81.6, 326.5, and 1306*μ*M, respectively. Statistical significance (P<0.05) was observed at 1306*μ*M concentration vs the negative control (OD=40.9 vs OD=32.3, respectively). Cells treated with H_2_O_2_ had an OD of 54.5, which was also statistically significant (P<0.05) when compared to the negative control. The OD of H_2_O_2_-treated cells was also statistically significant (P<0.05) when compared to all concentrations of fluconazole (Figure 5).

### 3.5. Evaluation of Apoptosis and Necrosis Using Differential Fluorescent Staining with Hoechst 33342/Propidium Iodide (PI)/Fluorescein Diacetate (FDA)

**Figure 6:**
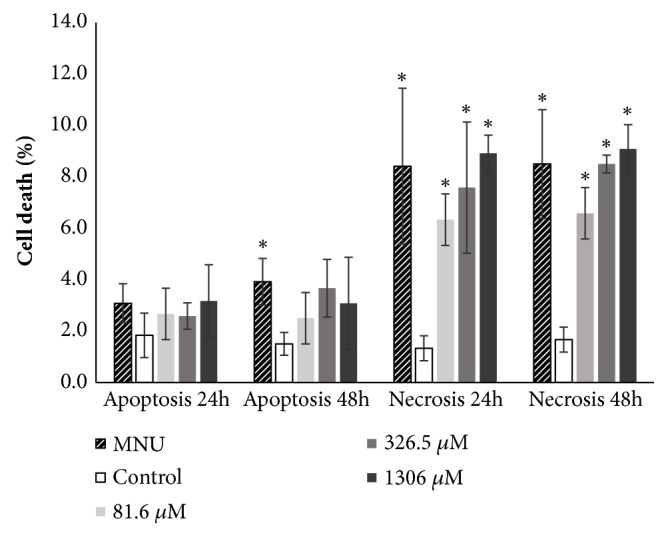
Effects of fluconazole in Vero cell line analyzed by differential fluorescent staining. *∗*P<0.05 (ANOVA/Tukey posttest and Kruskal–Wallis/Dunn posttest) when compared with control. Data are expressed as the mean values obtained from four experiments.

In our experimental conditions, fluconazole induced necrosis (P<0.05) in Vero cell line when cells were exposed to all concentrations (81.6, 326.5, and 1306*μ*M) for both tested harvest times (24 and 48 h) as compared with the negative control. NMU-treated cells also underwent significant apoptosis, when compared with the negative control, after 48 hours of treatment. Even though fluconazole was not able to induce apoptosis in our experimental conditions, a significant increase in this kind of cell death was observed when cells were exposed to NMU, as compared to the negative control, after 48 hours of treatment (Figure 6).

## 4. Discussion

Albeit fluconazole is widely used as an antifungal agent, studies on its genotoxicity/cytotoxicity are controversial. Therefore, this study aims to contribute to and increase the existing knowledge on such effects. Regarding cell viability, our results showed that fluconazole can induce a significant decrease in such endpoint in our experimental conditions (Figure 2). However, Rodriguez et al. [14] observed that fluconazole was not able to reduce cell viability in a primary culture system of rat hepatocytes through an MTT assay, though the authors showed that ketoconazole, which is another azole antifungal agent, was able to decrease cell viability through MTT assay, within a 25-200*μ*M concentration range. In their study, short-treated (0.5-6 hours) rat hepatocytes were exposed to lower concentrations of fluconazole (100-1000*μ*M), whereas, in our experiments, fluconazole concentrations of 81.6-2612.1*μ*M were administered for 24 hours. De Logu et al. [15] also tested the effects of fluconazole after a 72-hour treatment of Vero cells using MTT; surprisingly, they did not observe a decrease in cell viability even with concentrations as high as 1000 mg/mL (3265.08*μ*M). In some papers, fluconazole has demonstrated a clear cytotoxic effect, in the same way as what we have observed in our experiments, although such effect was less significant when compared to other azoles. For example, Somchit et al. [16] showed that itraconazole induced a higher cytotoxicity in rat hepatocytes* in vitro* through the lactate dehydrogenase (LDH) activity assay when compared to fluconazole. A lesser cytotoxicity induced by fluconazole, as compared to itraconazole, was also observed in the livers of rats upon exposure to either single or subchronic doses* in vivo* [17]. The mechanisms that lead to azole hepatotoxicity are largely unknown; however, it was observed that ketoconazole is susceptible to FMO (flavin-containing monooxygenase) attack on the N-1 position and subsequently leads to the production of an unidentified toxic metabolite [18, 19]. According to Somchit et al. [16], a similar mechanism may occur for itraconazole- or fluconazole-induced hepatotoxicity. FMO is also found in human kidneys which raises a concern with regard to nephrotoxicity from chemicals that undergo FMO-dependent bioactivation [20].

MTT assay detects variations in cell viability; however, it does not supply information about the mechanisms that lead to such variations. Therefore, other tests should be carried out to elucidate such mechanisms. In the present study, we used fluorescent dyes to detect the mechanisms that decreased cell viability as assessed with the MTT assay. Using such dyes, we observed that fluconazole was able to significantly induce necrosis in Vero cells (Figure 6). We were not able to find studies on fluconazole-induced cytotoxicity* in vitro*; however, the cytotoxicity induced by fluconazole in rat hepatocytes* in vitro* as assessed with LDH assay may be attributed to necrosis [16]. In necrosis, disruption of the cell plasma membrane results in extracellular release of cytoplasmic enzymes, including LDH, which is a stable enzyme that leaks in relatively high amounts during cell plasma membrane damage [21].

As already stated, reports on genotoxicity of fluconazole are controversial. One of such studies was carried out by Yüzbaşioğlu et al. [8]. They assessed the genotoxic effects of fluconazole using both* in vivo* (chromosome aberrations in mouse bone-marrow cells) and* in vitro* (chromosome aberration, sister-chromatid exchange, and micronucleus tests in human lymphocytes) systems. Their results showed that fluconazole was not clastogenic* in vivo*; however, an increase in all endpoints assessed* in vitro* was observed, which is comparable to the increase in MN rate as observed in our experiments (Figure 3). The authors observed MN increase with lower concentrations of fluconazole (25*μ*g/mL=81.6*μ*M and 50 *μ*g/mL=163.2*μ*M). In fact, some reports showed that lymphocytes are more sensitive to the effects of some drugs as compared to established cell lines [22]. Yüzbaşioğlu et al. [8] also observed that* in vitro* treatment with fluconazole was not able to change CBPI, which is in line with our results (Table 1). However, the cytostatic effect of other azoles was reported in other studies using more accurate techniques. For example, through flow cytometry and western blot analysis, Chen et al. [23] observed that ketoconazole was able to induce growth arrest in G0/G1 phase in three cancer cells (COLO 205, Hep G2, and HT 29), which is probably due to decrease in cyclin D3 and CDK4 proteins. Itraconazole showed similar effects in gastric cancer cells [24]. Fluconazole-induced MN was also observed in newborn pups after transplacental exposure [25, 26].

In the current study, we observed an increase in ROS induced by fluconazole in Vero cells (Figure 5). Induction of ROS by fluconazole was mainly reported and observed in fungal cells [27, 28]. However, other azoles induce ROS in mammalian cells as well. For example, ketoconazole induces hepatic injury in mice through ROS generation, specifically through the formation of hydroxyl radical, peroxynitrite, superoxide anion, and nitric oxide. Some authors stated that an increase in myeloperoxidase, which is a major component of azurophilic neutrophil granules, may be responsible for oxidative stress observed in their experiments [29]. Similar results were found by Sozen et al. [30], as they observed hepatic injury in mice that was accompanied by an increase in ROS generation induced by itraconazole. They also assessed DNA damage through comet assay, and they found that itraconazole was able to increase DNA damage, which is comparable to our results for fluconazole (Figure 4). It is known that oxidative stress induces DNA damage, as ROS reacts with DNA thus causing cleavage of DNA strands, DNA-protein cross-linking, and purine oxidation, which ultimately lead to breaks that may be assessed by the comet assay [31–33]. Therefore, it is likely that the increase in the rate of MN, together with the increase in DI, as observed through comet assay in the present study, may be due to the reactions of DNA damage-induced ROS in Vero cells. According to Yüzbaşioğlu et al. [8], fluconazole-induced genotoxicity in human lymphocytes* in vitro* may be due to bioactivation of CYP2E1, as chemical interactions with this enzyme produce free oxygen radicals.

ROS induction, together with FMO activity (as discussed above), may also explain the cytotoxicity observed in our experiments (Figure 6). ROS induces lipid oxidation that can lead to the loss of integrity of both plasma and intracellular membranes, such as lysosomes, leading to an intracellular leak of proteases and consequently resulting in necrosis [34]. ROS production is a stress stimulus known to contribute to both apoptosis and necrosis [34, 35]. Nevertheless, although fluconazole exhibits oxidative stress-inducing properties, it was not able to induce apoptosis in our experimental conditions. Moreover, fluconazole failed to induce apoptosis in human adrenocortical carcinoma H295R cells and its clone HAC15 when such cells were treated* in vitro* [36].

In brief, the results of this study showed that fluconazole induces cytotoxic and genotoxic alterations in Vero cells. It is likely that these effects may arise from the ability of fluconazole to be an oxidative stress inducer and/or the presence of FMO in such cells. The main concern related to our conclusions is the fact that the indiscriminate use of fluconazole in high doses for a long period of time could trigger carcinogenesis, since the accumulation of successive DNA errors may affect genes related to cell cycle control, such as tumor-suppressor genes and protooncogenes.

## Figures and Tables

**Table 1:** Cytokinesis-block proliferating index (CBPI) observed in Vero cell line after exposure to different concentrations of fluconazole.

**Cytokinesis-block proliferating index (CBPI)**					
**Experiment**	**NMU**	**Control**	**Fluconazole (** ***μ*** **M)**		
			**81.6**	**326.5**	**1306**
**1**°	1.222	1.442	1.268	1.396	1.424
**2**°	1.330	1.364	1.268	1.396	1.424
**3**°	1.280	1.240	1.420	1.340	1.270
**Mean**	1.277	1.349	1.319	1.377	1.373
**Standard deviation**	+/- 0.05	+/- 0.10	+/-0.09	+/- 0.03	+/- 0.09

*∗*P>0.05 (ANOVA). Mean of three experiments.

## Data Availability

The data used to support the findings of this study are included within the article.
